# Validation of the nutrient-rich foods index estimated by 24-h dietary recall method among adults in Henan province of China

**DOI:** 10.1017/S1368980022000465

**Published:** 2022-06

**Authors:** Junya Zhai, Baihui Ma, Quanjun Lyu, Lijun Guo, Pipasha Khatun, Rui Liang, Minghua Cong, Yongxia Kong

**Affiliations:** 1 Department of Clinical Nutrition, The Affiliated Cancer Hospital of Zhengzhou University, Henan Cancer Hospital, Zhengzhou 450008, People’s Republic of China; 2 Department of Nutrition, The First Affiliated Hospital of Zhengzhou University, Zhengzhou, People’s Republic of China; 3 Department of Nutrition and Food Hygiene, College of Public Health, Zhengzhou University, Zhengzhou, Henan, People’s Republic of China; 4 Department of Comprehensive Oncology, National Cancer Center/National Clinical Research Center for Cancer/Cancer Hospital, Chinese Academy of Medical Sciences and Peking Union Medical College, Beijing, People’s Republic of China

**Keywords:** Nutrient-rich food index, Diet quality, Nutrient profiling, Validation

## Abstract

**Objective:**

A family of nutrient-rich food (NRF) indices was validated against the mean adequacy ratio (MAR) and their associations with obesity were tested.

**Design:**

Cross-sectional study. NRF indices include nutrients to encourage ranging from 6–11 (protein; fibre; vitamin A, vitamin C, vitamin E and vitamin B_12_; Ca; Fe; K; Mg; Zn) and two nutrients to limit (saturated fat and Na), described as NRFn.2 (where *n* 6–11), based on reference amount of 100 g or 100 kcal using the NRF index family of algorithms. The percentage of variation in MAR (*R* ^2^) was the criteria of index performance. Logistic regression models were applied to predict the association between NRF index and obesity.

**Setting:**

Three communities in Zhengzhou city, Henan province, China.

**Participants:**

A total of 656 adults were recruited from Henan as the subjects.

**Results:**

The NRF9·2 index, based on nine beneficial nutrients and two nutrients to limit, using the algorithm based on sums and 100 kcal, had the higher *R* ^2^ values (*R* ^2^ = 0·232). The OR for overweight (defined by BMI) in the 4th quartile (Q4) *v*. the 1st quartile (Q1) of the NRF9·2 index was 0·61 (95 % CI = 0·37, 0·98) after multiple adjustments.

**Conclusion:**

NRF9·2 index using the algorithm based on sums and 100 kcal gave the best predicted model for diet quality. NRF9·2 index score was associated with overweight defined by BMI, but it was not associated with central obesity. The NRF9·2 index is a valid tool to assess the overall diet quality among adults in Henan province of China.

It is estimated that in the year 2016, 41 million people worldwide died of chronic non-communicable diseases, equivalent to 71 % of all deaths^(1)^; genetic predispositions, modifiable risk behaviours (such as tobacco use, harmful use of alcohol, physical inactivity and unhealthy diets) and environmental risks were the main underlying determinant factors. In the year 2019, globally dietary risks were responsible for 7·94 million deaths and 188 million disability-adjusted life years among adults^(2)^, implying that strategic plans for the improvement of diets at the population level are imperative and alarming.

Despite the amount of knowledge on the benefits of a nutritionally balanced diet to prevent non-communicable diseases, the prevalence of these diseases has been increasing. More and more researchers found that non-communicable diseases were associated with a high intake of energy-dense, nutrient-poor foods. Consuming energy-dense, nutrient-poor foods may increase the risk of high energy intake, marginal micro-nutrient intake and low serum concentrations of vitamins^(3)^. Such unbalanced diets are modifiable risk factors for the development of obesity^(4)^, non-alcoholic fatty liver disease^(5)^, metabolic syndrome^(6)^, bone unhealthy^(7)^ and others. Moreover, the dietary guidelines for Americans since 2005 stated that the basic food groups should contain a variety of nutrient-dense foods and beverages. Thus, more attention was paid to nutrient profiling models, which are intended to capture the nutrient density of food^(8,9)^. Nutrient profiling models calculate the percentage requirements for key nutrients in foods relative to the dietary energy that the foods provide^(10)^. The nutrient-rich foods (NRF) index is a crucial nutrient profiling model, which is based on 6 to 20 nutrients to encourage and on zero to 3 nutrients to limit. Each food was assigned a unique NRF score that reflects its total nutritional value per reference amount. Not limited to individual foods only, the NRF algorithms can be applied to food groups, meals, menus and total diets^(11)^. Diets with high NRF index score protect against central obesity^(12)^, higher BMI^(13)^ and mortality^(14)^. A family of NRF indices has been validated against the healthy eating index (HEI)^(15)^ and the Dutch healthy diet index (DHD)^(16)^.

Though China has achieved remarkable economic progress in recent years, yet, the diet has been undergoing an alarming transition with increasing intakes of more fat, meat and energy-dense, nutrient-poor foods^(17)^. Drewnowski^(11)^ proposed that the chosen models must be validated against independent measures of a healthy diet and, ideally, against health outcomes. Since the validity of the NRF index has not yet been established in Chinese adults, it is the objective of the current study to test several NRF indices scores for measures of a healthy diet among adults in Henan province of China and to explore the association between the NRF index and obesity indicators.

## Subjects and Methods

### Study design and population

Participants for the current analysis were from the cross-sectional study, which aimed to collect information on the diet, life styles and anthropometry of 656 adults aged 25–75 years in three communities in Zhengzhou city, the provincial capital of Henan province in China during the year 2020. In total, 912 individuals were invited, of which 785 agreed to participate. Individuals with incomplete data (missing data on 24-h dietary recalls (*n* 26), on anthropometry (*n* 28), on covariates (*n* 28)) and implausible energy intake (*n* 47)^(18)^ were excluded. The study was approved by the Institutional Review Board. Written informed consent was obtained from all subjects.

### Dietary and covariant assessment

Data were collected by a structured questionnaire and through two days non-consecutive 24-h dietary recalls. Structured questionnaires were designed, which contained three sections. The first section was pertinent to personal data and the second one was pertinent to life style such as smoking habit, sedentary time, nap frequency, physical activity and grip strength, while the last section was related to the dietary assessment. To help the respondents answer accurately, dietary intakes assessed by 24-h recalls were investigated face to face with the aid of food models. The average daily intakes of various foods and nutrients were analysed by nutrition calculator (NCCW software), which was calculated based on the China Food Composition Tables^(19)^.

### Anthropometric measurements

Weight, height, waist circumference (WC) and hip circumference (HC) were measured by experienced investigators using standardised procedures. Body weight (nearest 0·1 kg) and height (nearest 0·1 cm) were measured in duplicate by using an ultrasonic weight and height instrument, while the participants were barefoot and wearing light clothes only. WC and HC were measured to the nearest 0·1 cm using a flexible metric measuring tape with the individual in a standing position. WC was measured around the abdomen at the level of the umbilicus. HC was the maximum circumference of the hip.

### Evaluation of nutrient-rich food index scores

NRF index scores were based upon several nutrient profile models previously investigated by Drewnowski et al.^(20)^. The number of beneficial nutrients has ranged from 6 to 20, whereas the number of nutrients to limit has ranged from zero to three. Considering the limitation of the Chinese Food Composition Table^(21)^, the current study included eleven nutrients to encourage (protein, dietary fibre, vitamin A, vitamin C, vitamin E, vitamin B_12_, Ca, Fe, Mg, K and Zn) and two nutrients to limit (saturated fat, Na), described as NRFn.2 (where *n* 6–11). Thus, only NRF6·2, NRF9·2 and NRF 11·2 index were adopted in the current study.

NRF index scores in the current study were calculated based on per 100 g, per 100 kcal. The daily reference intakes of nutrients were based on the recommended nutrient intake or adequate intake (AI) of adults except for saturated fat, which was based on acceptable macro-nutrient distribution ranges (Table 1)^(21)^. The algorithms used to calculate the NRF index scores evaluated are listed in Table 2 ^(22)^.

**Table 1:** Chinese dietary reference intakes based on age and gender for calculating nutrient-rich food (NRF) index and mean adequacy ratio (MAR)

Nutrients	Male			Female		
	25-	50-	65-	25-	50-	65-
Energy (kcal)	2250	2100	2050	1800	1750	1700
Protein (g)	65	65	65	55	55	55
Dietary fibre (g)	25	25	25	25	25	25
Vitamin A (μgRE)	800	800	800	700	700	700
Vitamin C (mg)	100	100	100	100	100	100
Vitamin E (mg *α*-TE)	14	14	14	14	14	14
Ca (mg)	800	1000	1000	800	1000	1000
Fe (mg)	12	12	12	20	12	12
Potassium (mg)	2000	2000	2000	2000	2000	2000
Mg (mg)	330	330	320	330	330	320
Zn (mg)	12·5	12·5	12·5	7·5	7·5	7·5
Vitamin B_12_ (μg)	2·4	2·4	2·4	2·4	2·4	2·4
Saturated fat (g)	25	23·3	22·8	20	19·4	18·9
Na (mg)	1500	1400	1400	1500	1400	1400
Vitamin B_1_ (mg)	1·4	1·4	1·4	1·2	1·2	1·2
Vitamin B_2_ (mg)	1·4	1·4	1·4	1·2	1·2	1·2
Niacin (mgNE*,†)	15	14	14	12	12	11

* NE (niacin equivalence).

† NE (mgNE) = niacin (mg) + 1/60 tryptophan (mg).

**Table 2:** Overview of algorithms for the nutrient-rich food (NRF) index score

Model	Algorithm	Reference amount	Comment
NRn*			
NRn_100 g	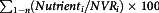	100 g	Nutrient_i_:content of nutrient i in 100-kcal edible portion NRV_i_ = Nutrient_i_ based on Chinese Dietary Reference Intakes
NRn_100 kcal	(NRn_100 g/ED) × 100	100 kcal	ED:energy density (kcal/100 g)
LIM†			Include saturated fat and sodium
LIM_100 g	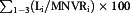	100 g	*L*_*i*_: content of limiting nutrient i in 100-kcal edible portion; MNRV_*i*_ :maximum daily values for nutrient i
LIM_100 kcal	(LIM_100 g/ED) × 100	100 kcal	ED: energy density (kcal/100 g)
NRFn.3			
NRFn.3_sum_l00 g	NRn_100 g- LIM_100 g	100 g	Difference between sums
NRFn.3_sum_l00 kcal	NRn_100 kcal- LIM_100 kcal	100 kcal	
NRFn.3_mean_l00 g	NRn/n_100 g- LIM/3_100 g	100 g	Difference between means
NRFn.3_mean_l00 kcal	NRn/n_100 kcal- LIM/3_100 kcal	100 kcal	
NRFn.3_ratio	NRn/LIM	None	NRn_100 g/LIM_100 g = NRn_100 kcal/LIM_100 kcal

* NRn = subscore based on a variable number n of beneficial nutrients.

† LIM = limited nutrient score.

### Assessment of nutrient adequacy

Nutrient adequacy was measured by computing mean adequacy ratio (MAR), an overall measure of the nutrient adequacy^(23)^. To compute MAR, nutrient adequacy ratio was first calculated for the selected ten nutrients (energy, protein, vitamin A, vitamin C, Ca, Fe, P, vitamin B_1_, vitamin B_2_ and niacin) as given in Table 1. Nutrient adequacy ratio was calculated based on Chinese Dietary Reference Intakes^(21)^. MAR was calculated as described by Madden et al^(23)^.

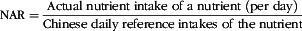




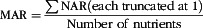




### Assessment of basic characteristics

BMI was calculated in the standard methods: weight (kg) divided by square of height (m), which was classified as underweight (< 18·5 kg/m^2^), normal weight (≥ 18·5 and < 23·9 kg/m^2^), overweight (≥ 24 and < 27·9 kg/m^2^) and obese (≥ 28 kg/m^2^) according to the Working Group on Obesity in China^(24)^.

Central obesity was defined by WC and waist:hip ratio (WHR). The cut-off point of WC was recommended by Working Group on Obesity in China: 85 cm for males and 80 cm for females^(24)^. WHR was calculated as WC (cm) divided by HC (cm). Central obesity was defined according to the WHO recommendation: WHR ≥ 0·90 for males and WHR ≥ 0·85 for females^(25)^.

Physical activity was collected through the Chinese version of the international physical activity questionnaire^(26)^, which appeared to have acceptable reliability and validity. The moderate-vigorous physical activity (MET-h/d, MET, metabolic equivalent of task) was calculated for each individual according to Chinese Guidelines for Chinese Residents^(27)^.

### Quality control

Quality control was carried out from questionnaire design to data analysis. First, the questionnaire used in the investigation was revised after pilot study and expert discussion. Second, all investigators must undergo training before the interview. Last but not the least, all data were inputted by two persons, and logical error detection and review were carried out.

### Statistical analysis

Data analysis was done by using SAS statistical software, version 9.3 (SAS Institute), for all data analyses. A *P*-value < 0·05 was considered statistically significant.

The distribution of variables was calculated and compared according to categories of NRF9·2 score based on the Kruskal–Wallis test for continuous variables. Multiple linear regression models were used to analyse the correlation between NRF index score and MAR, and the NRF index was selected according to the adjusted *R* ^2^. Potential confounders that were considered including age, gender, smoking (yes or not), life pressure (yes or not), grip strength (normal or not), sedentary time (h/d), family number and nap frequency. The NRF index score in quartiles (Q1–Q4) was taken as the independent variable and the dependent variable was overweight (including obesity) or central obesity. In the basic models (model 1), the correlation analyses between the NRF index score and overweight/central obesity were carried out first by crude OR with 95 % CI; model 2 was adjusted for age (continuous), gender and educational level (< 6, 6∼12, > 12 years). Model 3 was further adjusted for personal monthly income (< 2000, 2000∼5000 and > 5000 RMB), moderate-vigorous activity (low/relatively low/relatively high/high) and nap frequency (continuous).

## Results

### Validation

**Fig. 1:**
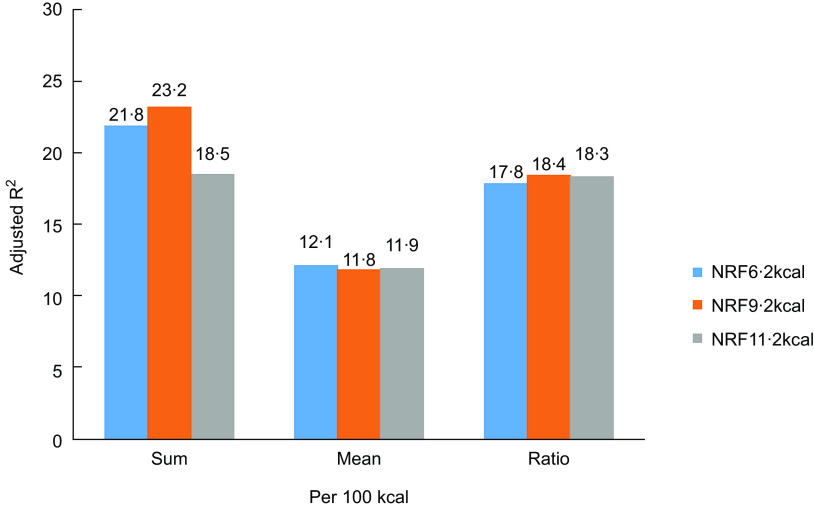
*R* ^2^ comparison of NRF n.2 algorithms calculated/100 kcal from regression models predicting MAR adjusted (*P* < 0·0001). NRF6·2, NRF9·2 and NRF11·2 (Details in the text)

**Fig. 2:**
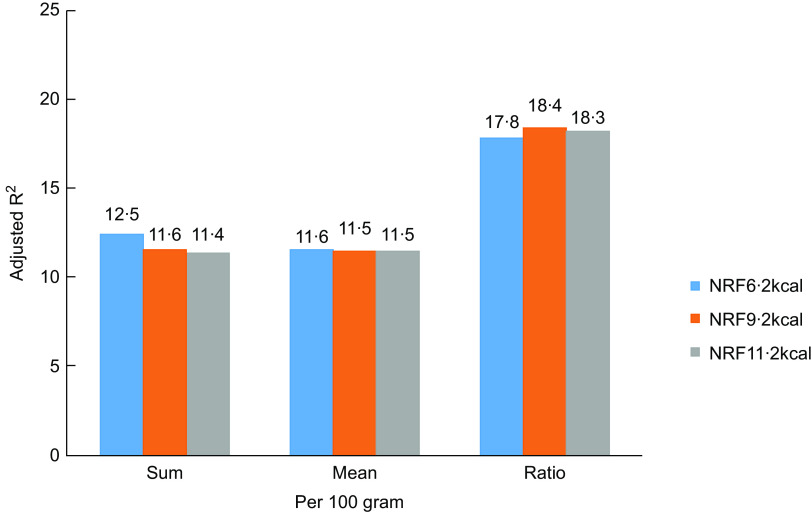
*R* ^2^ comparison of NRFn.2 algorithms calculated/100 gram from regression models predicting MAR adjusted (*P* < 0·0001). NRF6·2, NRF9·2 and NRF11·2 (Details in the text)

All NRF indices were positively correlated (*P* < 0·001) with MAR (Fig. 1 and 2), with adjusted *R* ^2^ ranging from 0·114 to 0·232 by adjusted for age, gender, BMI, smoking, life pressure, grip strength, sedentary time and family numbers. NRF9·2 index using the algorithm based on sums and 100 kcal had the highest *R* ^2^ values (*R* ^2^ = 0·232).

### General characteristics of the distribution of NRF9·2 index scores

Because the NRF9·2 index using the algorithm based on sums and 100 kcal had the best ability to predict MAR, we then scored all foods consumed by participants. The mean NRF9·2 index score of the participants was 39·93, 32·44 and 47·84 for the median, 25th and 75th quartile, respectively. We explore the distribution of NRF9·2 index score among gender, age, central obesity (defined by WC and WHR), overweight (defined by BMI), residence, personal monthly income, educational level, occupation, marriage and family numbers (Table 3). The diet quality of females (NRF9·2 index score was 41·37 (33·53, 50·73) for the median, 25th and 75th quartile) was higher than that of males (NRF9·2 index score = 38·83 (31·83, 46·25)). The diet quality of an individual with high education (NRF9·2 index score 43·33 = (36·30, 51·78)) was higher than that of a person with low education (NRF9·2 index score = 38·26 (32·37, 45·42)).

**Table 3:** General characteristics of the distribution of NRF9·2 scores

		The score of NRF9·2*		
Vairable	*n*	Median	25th percentile, 75th percentile	*P* value†
Total	656	39·93	32·44, 47·84	–
Gender				
Male	396	38·83	31·83, 46·25	0·004
Female	260	41·37	33·53, 50·72	
Age				
< 50	99	38·42	29·85, 47·78	0·350
50–65	407	39·91	32·49, 48·10	
> 65	150	41·04	34·19, 47·04	
Central obesity (WC)				
Yes	290	39·88	32·60, 47·96	0·6
No	366	40·04	31·71, 47·33	
Central obesity (WHR)				
Yes	298	39·68	32·62, 47·92	0·9
No	358	40·49	31·62, 47·87	
Overweight (BMI, WGOC)				0·7
Yes	285	39·90	32·75, 48·16	
No	371	40·30	31·97, 48·16	
Residation				0·067
Urban	559	40·43	32·58, 48·06	
Village	97	37·32	31·55, 44·03	
Personal monthly income				0·199
< 2000 RMB	152	38·26	32·37, 45·42	
2000–5000 RMB	399	39·91	31·74, 48·20	
> 5000 RMB	105	41·77	33·62, 48·99	
Educational level				< 0·0001
< 6 years	80	38·42	37·76, 55·66	
6–12 years	424	38·51	31·38, 45·39	
> 12 years	152	43·33	36·30, 51·78	
Occupation				0·13
Manual	98	37·43	31·69, 45·33	
Professional	82	41·61	33·45, 49·58	
Retired	332	40·23	33·11, 48·09	
Others	144	39·03	30·92, 47·21	
Marriage				0·84
Yes	601	39·94	32·46, 47·82	
Others	55	39·16	32·29, 49·53	
Family numbers				0·27
≤ 4	422	39·16	31·97, 47·50	
> 4	234	41·00	32·49, 48·13	

WC, waist circumference; WHR, waist:hip ratio WGOC, Working Group on Obesity in China.

* Values were presented as median (25th percentile, 75th percentile).

† The general characteristics of the distribution of NRF9.2 scores was tested by Kruskal–Wallis.

### Means of food groups and selected nutrients across quartiles of the NRF 9·2 index score

An inverse association was found between the NRF 9·2 and the consumption of cereals. At first quartile, with NRF 9·2 of 22·04 the respective mean cereals intake was 463·25 g, which was reduced down to 304·75 g in the fourth quartile with NRF 9·2 of 54·69. The estimated intakes of vegetables and fruits increased with the respective increase in NRF 9·2 index (Table 4). In terms of high-protein food intake, the overall intake was low, and the estimated intakes of milk, beans and egg increased with the respective increase in NRF 9·2 index, while there was no difference among the intake of meat, poultry and fish. We also found that the higher the NRF9·2 index score, the higher intake of nutrients to encourage. However, the intake of Se, Zn and phosphorous was not significantly associated with the NRF9·2 index score (Table 5).

**Table 4:** Means of food group intake across quartiles of the NRF 9·2 index score*

Food group	NRF9·2 index score†								*P* ‡
	Q1		Q2		Q3		Q4		
	Median	25th percentile, 75th percentile	Median	25th percentile, 75th percentile	Median	25th percentile, 75th percentile	Median	25th percentile, 75th percentile	
*n*	164		164		164		164		
NRF9·2 index score	22·04	26·43, 31·62	36·62	34·79, 38·09	43·28	41·72, 45·18	54·69	50·74, 59·57	< 0·0001
Cereal	463·25	318·20, 619·13	409·25	296·88, 520·00	373·90	271·63, 490·60	304·75	230·00, 416·35	< 0·0001
Vegetable	75·00	32·13, 129·80	128·00	67·55, 204·93	166·50	92·88, 249·75	208·50	102·75, 298·90	< 0·0001
Fruits	0	0, 172·00	3·5	0, 230·00	115·00	0, 299·55	250·55	101·85, 500·00	< 0·0001
Milk and milk products	0	0, 0	0	0, 0	0	0, 0	0	0, 9·38	0·006
Bean, nuts and seeds	0	0, 5	0	0, 12·63	0	0, 28·00	4·5	0, 29·70	< 0·0001
Meat, poultry and fish	23·60	0·00, 67·15	25·00	0·00, 68·75	25·00	0·00, 66·87	28·50	0·00, 82·78	0·29
Egg	20	0, 60	30	0, 60·00	41	0, 66·00	60	0, 70·23	0·004
Snacks§	8·00	2·00, 66·00	5·00	2·93, 11·90	5·00	3·00, 9·00	4·3	2·8, 8·18	0·057

* Values were presented as median (25th percentile, 75th percentile).

† Q1, 1st quartile; Q2, 2nd quartile; Q3, 3rd quartile; Q4, 4th quartile.

‡ The differences of food groups’ intake among quartiles of the NRF 9.2 index score were tested by Kruskal–Wallis test.

§ Snacks includes cookies, fast food, sugar preserved fruits and so on.

**Table 5:** Means of nutrients intake across quartiles of the NRF 9·2 index score§

Energy and nutrients	NRF9·2 index score*								
	Q1†		Q2†		Q3†		Q4†		
	Median	25th percentile, 75th percentile	Median	25th percentile, 75th percentile	Median	25th percentile, 75th percentile	Median	25th percentile, 75th percentile	*P* ‡
*n*	164		164		164		164		
Energy (kcal)	1560	1257, 2021	1470	1211, 1794	1380	1144, 1811	1384	1143, 1662	0·001
Fat (g)	39·25	28·68, 60·48	35·15	24·25, 50·55	34·10	23·92, 46·14	36·00	25·58, 44·90	0·008
Carbohydrate (g)	240·39	193·38, 305·66	245·67	195·97, 286·11	230·91	175·54, 287·14	215·52	170·47, 265·60	0·002
Vitamin B_1_ (mg)	0·63	0·45, 0·88	0·75	0·51, 0·97	0·75	0·57, 0·96	0·77	0·56, 0·97	0·018
Vitamin B_2_ (mg)	0·56	0·41, 0·74	0·58	0·46, 0·75	0·64	0·47, 0·83	0·72	0·54, 0·91	< 0·0001
Niacin (mg)	8·46	5·49, 12·12	8·96	6·10, 12·51	8·42	6·17, 11·88	9·73	7·14, 13·20	0·04
Phosphorous (mg)	702·55	556·48, 891·28	728·29	606·54, 937·15	764·80	623·57, 955·27	791·61	612·51, 958·30	0·06
Zinc (mg)	6·76	5·36, 8·46	7·26	5·89,8·94	7·06	5·74, 9·11	7·69	6·07, 9·51	0·095
Se (mg)	34·88	25·03, 49·95	38·48	28·33,50·81	38·96	27·30, 51·46	35·24	26·10, 50·43	0·6
MAR	0·53	0·44, 0·63	0·58	0·49, 0·66	0·62	0·52, 0·70	0·68	0·57, 0·77	< 0·0001

* Values were presented as median (25th percentile, 75th percentile).

† Q1, 1st quartile; Q2, 2nd quartile; Q3, 3rd quartile; Q4, 4th quartile.

‡ The differences of food groups’ intake among quartiles of the NRF 9.2 index score were tested by Kruskal–Wallis test.

§ This table listed the nutrients incorporated into the NRF9.2 index.

### The association between the NRF9·2 index scores and overweight, central obesity

The OR for overweight (defined by BMI) in the 4th quartile (Q4) *v*. the 1st quartile (Q1) of the NRF9·2 index was 0·61 (95 % CI = 0·37, 0·98) after multiple adjustments. However, the NRF9·2 index score was not related to central obesity, whether central obesity was expressed as WC or WHR (Table 6).

**Table 6:** The association between the NRF9·2 index scores and overweight/obesity indicators

Obesity indicators		NRF9·2 index score*						
			Q2		Q3		Q4	
		Q1	OR	95 % CI	OR	95 % CI	OR	95 % CI
Overweight (BMI, WGOC)	Model 1†	1·00	0·85	0·54, 1·33	1·12	0·72, 1·74	0·85	0·54, 1·33
	Model 2‡	1·00	0·87	0·55, 1·37	1·04	0·66, 1·65	0·73	0·46, 1·17
	Model 3§	1·00	0·76	0·47,1·22	0·92	0·57,1·48	0·61	0·37,0·98
Central obesity (WC)	Model 1†	1·00	0·85	0·52, 1·40	1·01	0·62, 1·65	0·85	0·52, 1·65
	Model 2‡	1·00	0·85	0·52, 1·40	0·97	0·59, 1·59	0·83	0·50, 1·37
	Model 3§	1·00	0·72	0·43, 1·22	0·94	0·56, 1·57	0·78	0·46, 1·33
Central obesity (WHR)	Model 1†	1·00	0·72	0·41, 1·26	1·05	0·62, 1·79	0·93	0·54, 1·59
	Model 2‡	1·00	0·70	0·39, 1·25	1·03	0·59, 1·79	0·93	0·54, 1·59
	Model 3§	1·00	0·60	0·32, 1·08	0·93	0·52, 1·65	0·97	0·54, 1·74

WGOC, Working Group on Obesity in China; WC, waist circumference WHR, waist:hip ratio.

* Values were presented as c.

† Crude model.

‡ Model 2 was adjusted for age (continuous), gender and educational level (< 6, 6–12, > 12 years).

§ Model 3 was further adjusted for personal monthly income (< 2000, 2000–5000, > 5000 RMB), nap frequency (continuous) and moderate-vigorous activity (low/relatively low/high/high).

## Discussion

The NRF index has been proposed to predict overall diet quality in Americans, Dutch and Japanese, while it has not yet been evaluated in Chinese. In the current study, we observed that the optimal NRF indices was the NRF9·2 index, which was composed of nine nutrients (protein; fibre; vitamin A, vitamin C and vitamin E; Ca; Fe; K and Mg) to encourage and two nutrients (saturated fat, Na) to limit, using the algorithm based on sums and 100 kcal among adults in Henan province of China. The NRF9·2 index score was found to be related not only to the foods/food groups but also to other essential nutrients not incorporated into the NRF9·2 index, such as thiamine, riboflavin, nicotinic acid, phosphorus and Zn. NRF9·2 index was inversely associated with overweight (BMI, Working Group on Obesity in China), but not with central obesity after adjustment for potential confounders. These results revealed that the NRF9·2 index can be used as a valid tool to assess the overall diet quality among adults in Henan province of China.

Choosing the best NRF index among multiple alternatives is a scientific challenge. Of the fifteen tested scores, the prediction of the MAR was highest for the NRF9·2, with an *R* ^2^ of 0·23. In the previous study, the NRF9·3 index based on 100 kcal best predicted the HEI-2005 with an *R* ^2^ of 0·45^(15)^ and the DHD-index with an *R* ^2^ of 0·34^(16)^. Compared with the above studies, the proportion of explained variance of the NRF index scores against the MAR was somewhat lower, but not to a great extent. This might be caused by the different daily reference intakes of nutrients, different study populations, differences between MAR and the HEI and the DHD-index or different nutrients included in the NRF index. Considering the less readily available added sugars data and the relatively low consumption level of added sugar in China^(28)^, the total sugar or added sugar is not incorporated into the NRF indices. In addition, the current study confirmed previous studies^(20)^ showing that increasing the number of nutrients above 10 in a nutrient profile model provided little or no additional benefit in predicting overall diet quality. This choice was mainly based on Americans, whereas other nutrients might be more important for certain specific health outcomes or the Chinese. Nevertheless, the prediction of the MAR did not differ to a great extent between the scores and NRF index performed best in the Chinese population as well as in the USA and Dutch population, a nutrient profile model for specific nationality from other parts of China and for a special purpose is expected. Algorithms per 100 kcal, best reflected the original concept of nutrient density of foods, had higher *R* ^2^ values than those based on 100 g which makes no allowances for the fact that different foods and beverages are consumed in very different amounts^(11)^. The preferred algorithms were those that were based on sums, rather than a mean or ratio between the positive and negative nutrients. Compared with algorithms based on mean or ratio, those based on sums appear to be simplest, more transparent and weigh all nutrients equally^(29)^.

In the current study, the participants with the higher NRF9·2 scores had lower intakes of cereals and snacks, while with higher intakes of vegetables, fruits, milk, beans and eggs; in terms of nutrient, the higher NRF9·2 scores, the higher intakes of vitamin B_1_, vitamin B_2_ and niacin, the lower intakes of energy, carbohydrate and fat. Therefore, the NRF9·2 index can be used as one of the effective tools to evaluate dietary quality from the point of view, as it is consistent with the key recommendations of dietary guidelines for Chinese residents^(27)^. While the overall high-protein food intake of this population was low, the phenomenon may be related to the dietary survey obtained via 24-h dietary recalls, and the eating habits mainly based on cereals and cereal-based foods, vegetable, fruits and others. While socio-economic factors correlated with the NRF9·2 index were identified. Females and participants who had a higher level of education had better diet quality, which is consistent with the existing dietary indices^(30)^. This is likely due to increased nutrition awareness^(31)^, which is consequently translated into better dietary practices.

At present, multiple efforts to explore the relationship of nutrient profile models and various measures of anthropometry are underway. It is, however, not yet clear whether the NRF index is helpful in weight management. A study on 2696 adults from the USA and the UK^(32)^ showed that the NRF index was negatively related to BMI, which was consistent with our findings. And a study in Egyptian youths showed that NRF9·3 index was correlated negatively with markers of abdominal obesity^(33)^, which was different from our results, while a study consisting of 4969 Dutch participants aging > 55 years reported positive correlation between NRF index score and BMI^(34)^, WC and WHR^(34)^. Causative factors for this discrepancy include underreporting of food intake among the obese participants, unique different characteristics of the participants such as race, age, gender and health status; different cut-off points of obesity; different methodologies of statistical analysis and adjustment of possible confounding factors; variation in the definition of some food ingredients such as differentiation between added sugars^(32)^ and total sugars^(34)^.

The present study has its limitations. First, our study had a cross-sectional design, which failed to determine the exact causality of NRF9·2 index and weight gain, and should be interpreted cautiously. Therefore, we plan to conduct a follow-up study to explore the cause–effect relationship. Second, the finding was only applicable to adults in Henan province of China, as China has a vast territory and abundant resources, and there were great cultural differences among different ethnic groups. More studies are needed to be carried out on different ethnic groups from different Chinese regions. Therefore, we plan to conduct multi-centre research to increase the representativeness of the sample. The other limitation of this research was that it did not take into account other beneficial nutrients or other non-nutrient substances like phytochemicals, which may be essential for the Chinese. Finally, the sample used in our analysis was not as large as that used in other cross-sectional studies. However, our analysis excluded any energy under-reporters and was carefully adjusted with potential confounders.

## Conclusion

To our knowledge, we are the first who studied the validation of the NRF index in Chinese adults. Our findings demonstrated that the NRF9·2 index, using the algorithm based on sums and 100 kcal, was the best predicted model with high association with MAR and with BMI, and rendering this index the best predicted model and valid tool to assess the overall diet quality among adults in Henan province of China. Modifying food-selected behaviour through consuming a nutrient-dense diet may be an important approach to control epidemic obesity.
